# Performance Prediction of Erosive Wear of Steel for Two-Phase Flow in an Inverse U-Bend

**DOI:** 10.3390/ma15165558

**Published:** 2022-08-12

**Authors:** Saifur Rahman, Rehan Khan, Usama Muhammad Niazi, Stanislaw Legutko, Muhammad Ali Khan, Bilal Anjum Ahmed, Jana Petrů, Jiří Hajnyš, Muhammad Irfan

**Affiliations:** 1 Electrical Engineering Department, College of Engineering, Najran University, Najran 61441, Saudi Arabia; 2 Department of Mechanical Engineering, College of Electrical and Mechanical Engineering, National University of Sciences and Technology, Islamabad 44000, Pakistan; 3 Department of Mechanical Engineering Technology, National Skills University, Islamabad 44000, Pakistan; 4 Faculty of Mechanical Engineering, Poznan University of Technology, 60-965 Poznan, Poland; 5 Department of Machining, Assembly and Engineering Metrology, Mechanical Engineering Faculty, VŠB-Technical University of Ostrava, 17. Listopadu 2172/15, 708 00 Ostrava, Czech Republic

**Keywords:** erosion, wear, U-bends, discrete phase model, sand, elbow

## Abstract

Erosion of the elbow due to non-Newtonian viscous slurry flows is often observed in hydrocarbon transportation pipelines. This paper intends to study the erosion behavior of double offset U-bends and 180° U-bends for two-phase (liquid-sand) flow. A numerical simulation was conducted using the Discrete Phase Model (DPM) on carbon steel pipe bends with a 40 mm diameter and an R/D ratio of 1.5. The validity of the erosion model has been established by comparing it with the results quantified in the literature by experiment. While the maximum erosive wear rates of all evaluated cases were found to be quite different, the maximum erosion locations have been identified between 150° and 180° downstream at the outer curvature. It was seen that with the increase in disperse phase diameter, the erosive wear rate and impact area increased. Moreover, with the change of configuration from a 180° U-bend to a double offset U-bend, the influence of turbulence on the transit of the disperse phase decreases as the flow approaches downstream and results in less erosive wear in a double offset U-bend. Furthermore, the simulation results manifest that the erosive wear increases with an increase in flow velocity, and the erosion rate of the double offset U-bend was nearly 8.58 times less than the 180° U-bend for a carrier fluid velocity of 2 m/s and 1.82 times less for 4 m/s carrier fluid velocity. The erosion rate of the double offset U-bend was reduced by 120% compared to the 180° U-bend for 6 m/s in liquid-solid flow.

## 1. Introduction

Erosion of pipeline components can cause serious malfunction for the hydrocarbon extraction and processing industries, as the sand produced can impact the walls of the pipeline, resulting in wear damage [1,2]. When the dispersed phase has to be transported, flow-changing devices, i.e., elbows, are inclined to erosion damage because of the redirected flow at curvature. Most of the available experimental and numerical data focus on long radius 90° elbow erosion [3,4,5]. In erosive wear modeling, the understanding of flow physics around the erodent and the target surface is crucial. Many researchers investigated to understand the flow of physics during particle wall interaction. The particle trajectories and the erosion rate are strongly affected by the momentum exchange between flowing fluid and solid particles [6,7,8,9,10].

Elemuren et al. [11] investigated 90° elbow erosion by employing experimentation. It was noticed from the surface topographies of the elbow upstream, middle and downstream sections that ridges and valleys are discerned downstream of the elbows at higher particle loadings.

X. Zhao et al. [12] performed numerical prediction on the wear of bends installed in a series configuration in the two phase (gas-solid) flow. The result of the study shows a V-shaped erosion pattern due to the secondary impaction of the disperse phase at outer curvatures. Additionally, they found that the erosion of downstream bends is significantly influenced by the upstream pipe due to the considerable change in particle impaction velocity and angle.

Q. Wang et al. [13] simulated erosion in elbow geometry using large eddy simulation (LES). It was observed that the highest erodent impaction remains at the outer wall of the elbow outlet. Moreover, the maximum erosion rate decreases as the bend curvatures increase.

Wang et al. [14] utilized the CFD approach to predict the erosion of a 90° elbow. It was noticed that the erosion distribution is also influenced by the erodent size; the maximum impaction and erosion location will be located near the elbow outlet. The location of maximum impaction shifted close to the exit section with the increase in particle size.

Khan et al. [15] investigated multiphase erosion flow for bend angles (60° and 90°) with sand particles using a flow loop. They observed that as the elbow angle increases, the erosive wear increased for the 90-degree pipe bend, and the erosion rate decreases as bend angles decrease. It was also found that the maximum erosion location will be at the downstream section for both elbow configurations. Duarte et al. [16] decoupled the relationship between sand erodent concentrations and erosive wear for elbow pipes by employing CFD. It was observed that the influence of interparticle collisions on the erosion rate is significant for low mass loading conditions, and erosive wear decreases with an increase in mass loading due to particle–particle interactions. Karimi et al. [17] predicted sand fines erosion of a 90-degree elbow utilizing numerical simulations. They found that CFD inaccurately predicts erosion due to sand fines in the elbow configuration; however, for direct impact cases, CFD results show good agreement with experiments. In elbow configuration, the rebound models were not simulating the fine particle trajectories inside the pipe correctly.

Bilal et al. [4] studied the influence of pipe bend R/D ratio on the wear rate and concluded that the erosive wear rate in multiphase flow is larger than that of single-phase flow conditions. Cui et al. [18] conducted a computational fluid dynamics simulation to quantify particle erosion in the elbow pipe for the bubble flow regime.

Li et al. [19] calculated erosion for continuous elbows in different directions by utilizing CFD-based simulation. It was noticed that the erosion rates of 50-micron particles are larger than those of the 10-micron particle size for the identical gas flow rate. H. Zhu and Y. Qi [20] numerically investigated the flow erosion of multiphase flow in a U-bend. They found that flow velocity and particle size significantly influence erosion rate and lead to excessive erosion. Mazumder [21] performed a numerical and experimental investigation on S-bend geometry erosion in multiphase flow to identify the location of erosion inside the pipeline.

In erosive wear modeling, the understanding of flow physics around the erodent and the target surface is crucial. Many researchers investigated erosive wear to understand the flow of physics during particle wall interaction. The particle trajectories and the erosion rate are strongly affected by the momentum exchange between flowing fluid and solid particles [6,7,8,9]. In gas-solid flows due to the high inertia of erodent, they cross the flow streamlines, while in liquid-solid flow the drag forces on erodent are higher due to high viscosity. Numerical predictions of impact conditions require many assumptions to quantify erosion rate, but CFD has been a widely adopted method to predict the impact condition in the fluid flow [22,23,24]. The most important factor that strongly influences the accuracy of CFD erosion prediction in liquid-solid-gas flow is particle distribution and size in carrier phases. For erosion-induced wear, hydrodynamics plays an important role. Numerous flow dynamics parameters due to slurry (liquid-solid) transport through 90° elbow configurations were numerically simulated and experimentally investigated in previous studies, and erosion prediction models were developed to quantify the erosion-induced damage for elbow configurations [25,26].

Previous studies on the erosion of elbow pipelines have mainly focused on 90° elbows. To the best of our knowledge, studies related to double offset U-bend and 180° U-bend erosion in liquid-solid flow have not been reported in the literature. In the hydrocarbon production industry, there are cases where elbow pipes were suspected to have an erosional impact on production systems, especially in transient operations. Prediction of such an effect will be beneficial to material selection, wall thickness design as well as erosion mitigation methods.

In this paper, the erosion of inverse double offset U-bend and 180° U-bend is studied for different flow velocities and particle sizes using a CFD-DPM. To validate the simulation model and flow physics, the predicted erosive wear of an elbow was compared with the data obtained by Mazumder [21] and W. Peng, X. Cao [27].

## 2. Problem Description

### 2.1. Model Geometry

**Figure 1:**
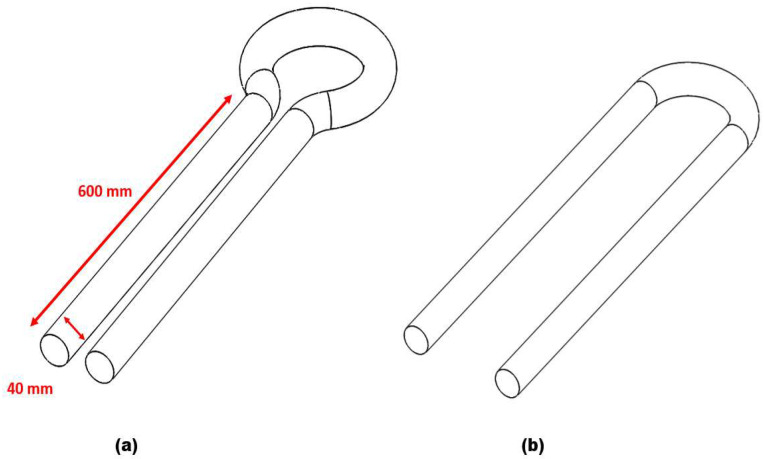
Elbow geometry: (**a**) double offset U-bend; (**b**) 180° U-bend.

Figure 1a,b is a geometry of a double offset U-bend and a 180° U-bend used as a computational domain in this study. The double offset U-bend and 180° U-bend with an internal diameter (ID) of 40 mm and an R/D ratio of 1.5 are selected in the research. The entry and exit pipe of the bend geometry is 600 mm to ensure the fully developed flow in the upstream pipe. The elbows are made of carbon steel and the orientation is inverse (vertical upward–vertical downward).

### 2.2. Carrier and Solid Phase Model

In this study, the numerical equations of the FLUENT module of ANSYS used to solve the liquid-solid flow physics are represented as Equations (1) and (2):

(1) $$\frac{\partial \rho }{\partial t}+\nabla \cdot \rho \vec{V}=0$$

(2) $$\frac{\partial }{\partial t}(\rho \vec{V})+\nabla \cdot \rho (\vec{V}\vec{V})=-\nabla P+\nabla \cdot (\overset{=}{\tau })+\rho \vec{g}+\vec{S_{M}}$$

(3) $$m_{e}\frac{d{\vec{u}}_{p}}{dt}={\vec{F}}_{{}_{1}}\;+{\vec{F}}_{{}_{2}}+{\vec{F}}_{{}_{3}}+{\vec{F}}_{{}_{4}}$$

In Equation (2), *ρ* = density, *τ* = stress tensor, *ρ*g = body force, and *S_M_* = momentum. The disperse phase model can be expressed in Equation (3), where *m_p_* = erodent mass, *F*_1_ = drag force, *F*_2_ = pressure force, *F*_3_ = particle force, and *F*_4_ = buoyancy force.

### 2.3. Erosion and Turbulence Model

The erosion model defined by Oka is used in this study to quantify the erosion distribution of the double offset U-bend and the 180° U-bend, which is the reasonably accurate erosion prediction model of curve pipes [1] and is defined as:

(4) $$ER=10^{9}\times \rho_{t}kF(\alpha ){\left(H_{v}\right)}^{k_{a}}{\left(\frac{V_{e}}{V^{\prime }}\right)}^{k_{b}}{\left(\frac{d_{e}}{d^{\prime }}\right)}^{k_{c}}$$

In Equation (4), *ρ_t_* = density of wall, *α* = sand incidence angle, *H_v_* = Vickers hardness of wall, *V_e_* = incidence speed, *V*′ = reference speed, *d_e_* = sand size, and *d*′ = reference sand size. In this study, the Grant and Tabakoff model [28] is selected to model particles and wall collision. The turbulence model (k–ε) was selected in this study since the Reynolds number calculated is larger than 4000.

### 2.4. Mesh and Model Validation

**Figure 2:**
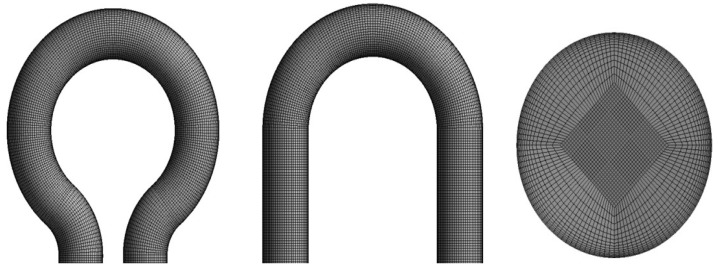
Computational mesh.

As shown in Figure 2, hexahedral structured meshing was generated on the U-bend configurations with approximately 478,000 cells. The boundary layer grids with 10-layer were implemented near the wall to capture the flow field structure near the wall. The flow solution chosen for this study is steady-state with 10^−6^ of the convergence criterion and the SIMPLEC numerical procedure is used to discretize the multiphase phase flow.

**Figure 3:**
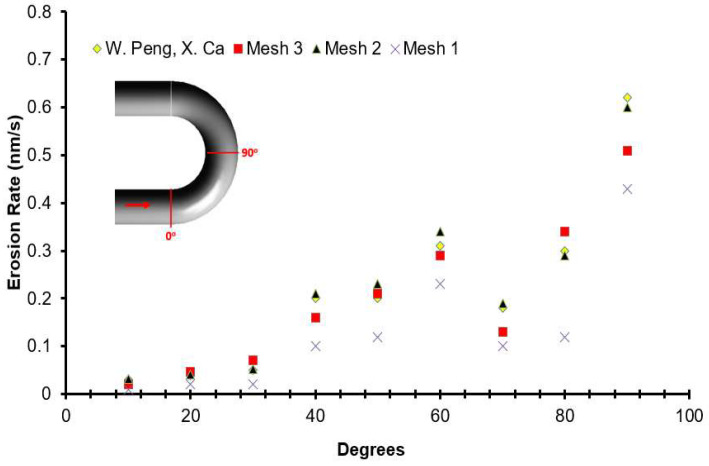
Comparison of the erosion rate obtained with three different mesh sizes and numerical results in W. Peng, X. Ca [27].

To further improve the simulation accuracy, the mesh independence study was performed. The validation study was performed by comparison with the maximum rate of erosion with the benchmark case of W. Peng and X. Cao [27]. The flow conditions are set the same in comparison with the benchmark study. It can be concluded that, for this geometry, most of the erosion is predicted at the outlet location irrespective of the type of mesh. The erosion rate was evaluated from three meshes of different sizes, as presented in Figure 3. It can be seen that there is less than a 2% difference in the results between mesh 2 and mesh 3, and the numerical simulations for the present investigation was conducted using mesh 2 with 478,000 cells. Fourteen cases with carrier fluid water (W) and Air (A) are scrutinized, as summarized in Table 1.

**Figure 4:**
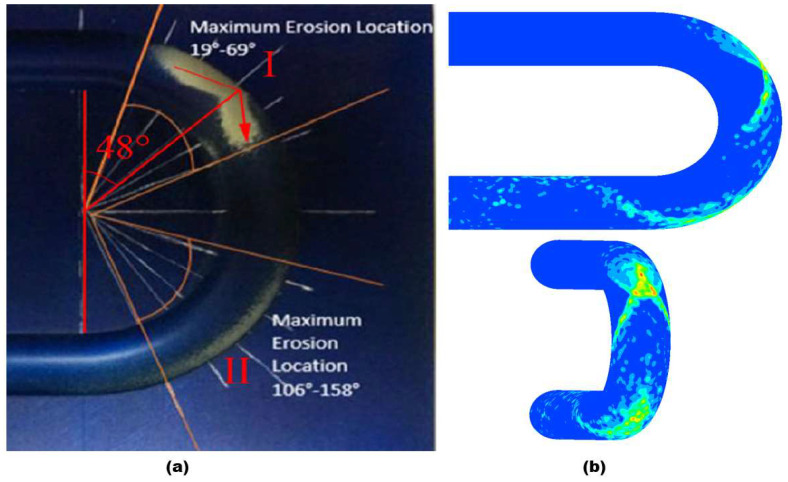
Comparison of the erosion location: (**a**) experimental results in Mazumder [21]; (**b**) numerical results (present study).

A validation case was simulated to adjudge the effectiveness of the numerical model and particle tracking algorithm used in this study. The same parameters as the experiment of Mazumder [21] were set in the validation case. Air-solid flow enters a U-bend (ID = 12.7 mm, r/D = 1.5) at a velocity of 45.7 m/s, and 300 microns sand size. The comparison of the erosive wear location between the simulation and experiment at the outer wall is shown in Figure 4. The results obtained by CFD-DPM are consistent with the experiment with the first location of erosion observed at 19–69° and the second observed at 106–159°. As seen in Figure 4, the CFD identified accurately the erosion location that occurs at the outer wall as compared to the qualitative experiment.

## 3. Results and Discussion

### 3.1. Effects of the Flow Velocity on Erosion

**Figure 5:**
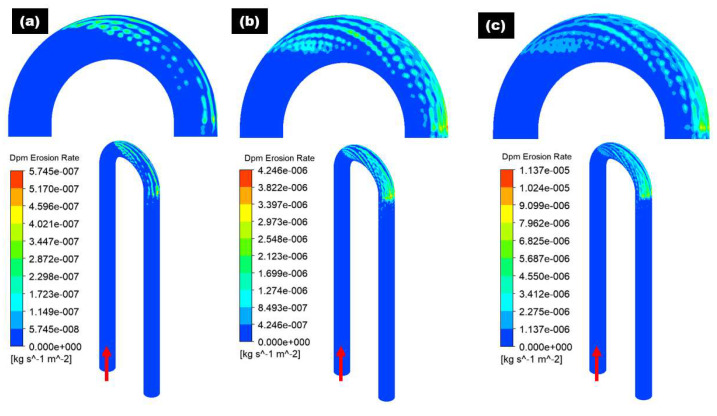
Erosion distribution in 180° U-Bend: (**a**) 2 m/s; (**b**) 4 m/s; (**c**) 6 m/s.

**Figure 6:**
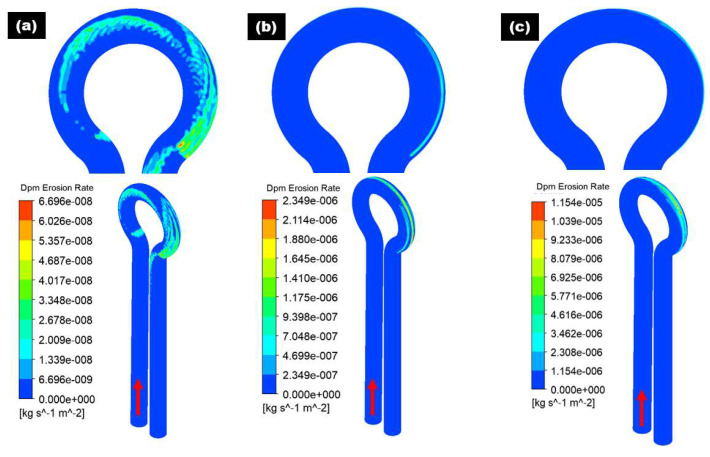
Erosion distribution in double offset U-bend: (**a**) 2 m/s; (**b**) 4 m/s; (**c**) 6 m/s.

**Figure 7:**
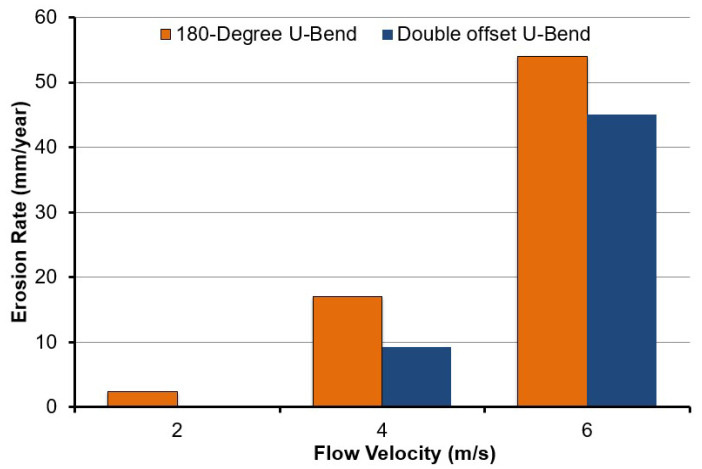
Maximum erosion rate in 180° U-bend and double offset U-bend for three flow velocities.

To decouple the influence of flow velocity on erosive wear distribution in the 180° U-bend and double offset U-bend, to avoid settling of erodent particles by maintaining the carrier flow velocities above certain levels in the pipelines, the cases with inlet velocity set to 2, 4 and 6 m/s are considered. The carrier fluid is water and the length of the pipe before and after the bend is set to 600 mm. Figure 5 and Figure 6 show the erosive wear distribution contours on the 180° U-bend and double offset U-bend for different flow velocities. It can be discerned that the maximum erosive wear remains located in the outlet for all inlet velocities, and the maximum erosion rate of both the 180° U-bend and double offset U-bend increases drastically with the accretion in flow velocity. As presented in Figure 5, at the lowest flow velocity of 2 m/s, the highest erosion rate of the 180° U-bend locates adjacent to the exit of the bend. With the accretion of the fluid velocity, the exit of the bend at the outer curvature is significantly eroded and becomes the highest impaction location when the flow velocity reaches 6 m/s. The most severely eroded location of the double offset U-bend is shown in Figure 6, the simulation predicts a reduction of maximum erosion rate by a factor of 8.58 from Case 1 to Case 4 because more sand impaction occurs at the outer elbow curvature of Case 1 as opposed to Case 4. Furthermore, for Case 2 and Case 5, the decrease in the maximum erosion rate was about 1.81 times. As the redirection of flow is smoother for double offset U-bend, more sand follows the fluid stream, and less sand impaction occurs at the outer elbow curvature. For similar cognitions, Case 6 eroded less compared to Case 3, as shown in Figure 7.

The erosion contour changes from symmetric to a more concentrated outer wall with the change from the 180° U-bend and double offset U-bend, and the zone affected by sand erosion is the minimum.

**Figure 8:**
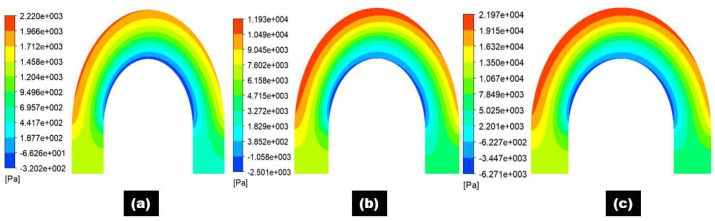
Pressure distribution contours of 180° U-bend for three different flow velocities: (**a**) 2 m/s; (**b**) 4 m/s; (**c**) 6 m/s.

**Figure 9:**
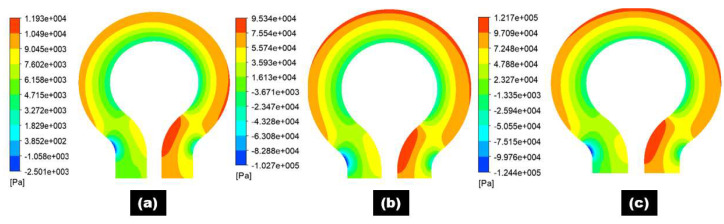
Pressure distribution contours of double offset U-bend for three different flow velocities: (**a**) 2 m/s; (**b**) 4 m/s; (**c**) 6 m/s.

**Figure 10:**
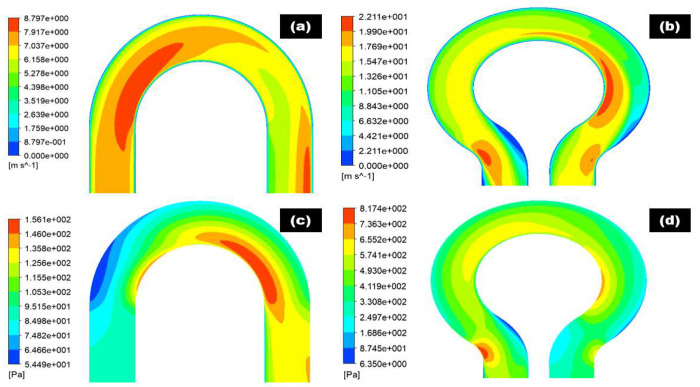
Contours of 180° U-bend and double offset U-bend for 6 m/s flow velocities: (**a**,**b**) velocity distribution; (**c**,**d**) wall shear stress.

Figure 8 and Figure 9 show the pressure distribution at selected planes inside the 180° U-bend and double offset U-bend. Simulations were undertaken with input velocities of 2, 4, and 6 m/s for the 180° U-bend and double offset U-bend. The centrifugal force acted on the fluid and was impelled to the outer curvature of the bend, which was exposed to higher pressure, while the inner curvature was subjected to lower pressure. However, as an example, only two extreme velocity distributions in the 180° U-bend and double offset U-bend are shown here for the case with the maximum erosion rate in Figure 10a,b. It is evident from these figures that the changing geometry from the 180° U-bend to the double offset U-bend can significantly influence the flow field. As the input velocity increased from 6 to 8.8 m/s in the 180° U-bend and from 6 to 22 m/s in the double offset U-bend, the flow pattern changed significantly, especially at the curvature of the 180° U-bend with a 1.46 times increase in velocities. In contrast, the input velocity accreted from 6 to 22 m/s in the double offset U-bend with a 3.66 times increase in velocities.

Wall shear stresses inside the 180° U-bend and double offset U-bend under 6 m/s velocities of carrier fluid were simulated by CFD-DPM as shown in Figure 10c,d. The highest wall shear stress is observed at the inner curvature in the 180° U-bend and outer curvature in the upstream pipe of the double offset U-bend and then the second location of maximum wall shear located in the inner curvature of pipe in the double offset U-bend. When the carrier fluid is transported into the bend pipe, the fluid direction is altered and the pressure of the fluid on the outer wall of the bend pipe enhances under the action of centrifugal force. The carrier fluid kinetic energy on the outer curvature results in the pressure, and the carrier fluid velocity on the outer curvature of the bend reduces. Consequently, the reduction in wall shear stress at outer curvature is observed as shown in Figure 10c,d.

### 3.2. Effect of Sand Size on Erosion

**Figure 11:**
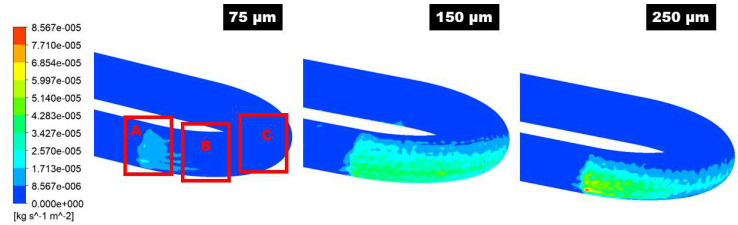
The contour of the erosion distribution in the 180° U-bend elbow under three different particle sizes.

**Figure 12:**
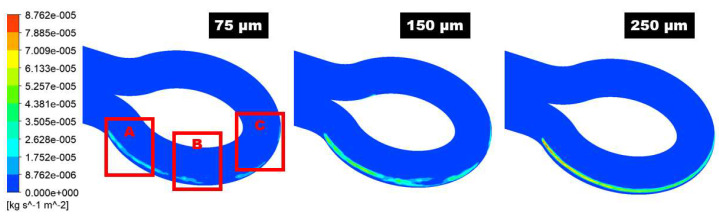
The contour of the erosion distribution in the double offset U-bend elbow under three different particle sizes.

The maximum sand impaction zones will dynamically change with the change of particle diameter in the 180° U-bend and double offset U-bend in Figure 11 and Figure 12. Zone A is inclined to maximum erosive wear when the particle diameter is 75 µm as shown in Figure 11. Consequently, as particle diameter increases, this maximum particle impaction will remain in Zone 1 with the addition of a medium erosion zone in the downstream section. When the particle size is 75 µm, the drag force is prepotent, and the transportation of sand is due to secondary flow. The flow pushes the sand in the circumferential path from the curvature to the exit of the bend and then moves the sand to the exit of the bend’s outer curvature and, as a result, causes maximum erosion in Zone A. For 250 µm sand, the inertia force is a significant parameter in the transportation of the sand. The sand erodent has high momentum; thus, the flow velocity and direction have a slight effect on the sand. Since the sand particles divagate from the flow streamlines, impaction occurs at Zones A, B, and C. It is noticed that for all the evaluated case, the one maximum erosion zone appears along the curvature of the 180° U-bend. Nevertheless, the erosive wear becomes more serious as the particle size accrete. For 75 µm, erosion spots are located at Zone A at the outer curvature. As the sand size changes from 75 to 250 µm, the area of erosion location is significantly increased and hence leads to maximum erosion. Additionally, the severe erosion in the exit elbow section becomes visible for the sand size of 250 µm. The width of the maximum eroded region grows as the size of sand further increases.

**Figure 13:**
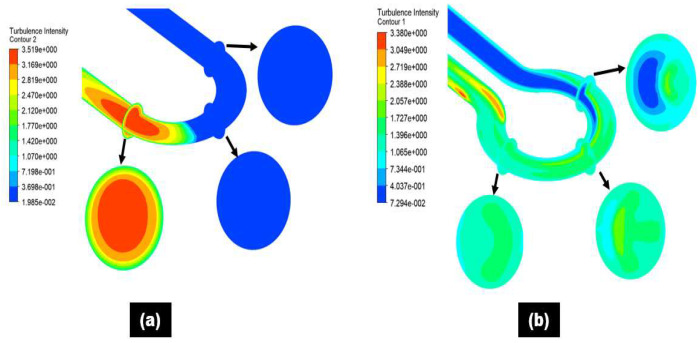
Turbulence intensity at the different locations inside the elbow pipe for 250 µm particle size: (**a**) 180° U-bend; (**b**) double offset U-bend.

Figure 13 shows that turbulence intensity increases sand impaction impact and erosive wear at the curvature near the outlet of the 180° U-bend and double offset U-bend. The turbulence in the fluid stream at the outlet of both bend configurations enhances the motion of sand in the radial directions and turns out to be a maximum erosive zone. This signifies that turbulence intensity causes the shift of particle trajectories inside the 180° U-bend and double offset U-bend. Based on analysis, turbulence weigh more in the 180° U-bend at the downstream as compared to the double offset U-bend. Figure 13 shows the contours of turbulent intensity in the entry, middle, and exit of the 180° U-bend and double offset U-bend. Figure 13 suggests that the reduction in the blue area and the enhancement in the red and yellow area signify the increase in turbulence. This means that maximum turbulence turns out in the 180° U-bend elbow exit sections.

**Figure 14:**
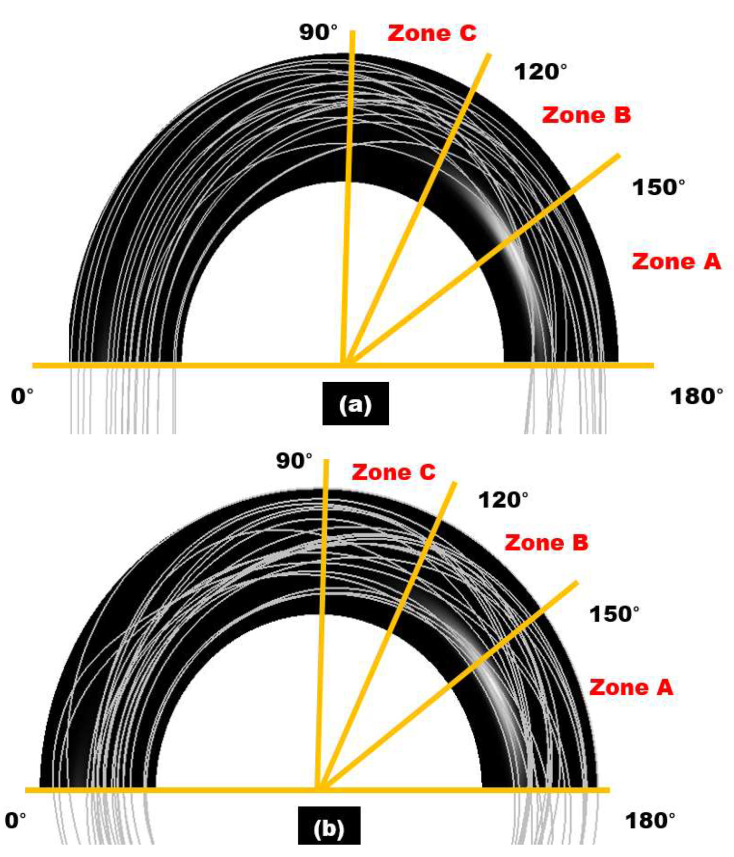
Trajectories of 250 µm sand particles under the particle velocity of 6 m/s: (**a**) 180° U-bend; (**b**) double offset U-bend.

Figure 14a,b show the trajectories of selected sand within the 180° U-bend and double offset U-bend. When the sand is 250 µm, the turbulence leads to the sand distribution and sand particles distribute evenly in the upstream pipe in a 180° U-bend. With the change of configuration from the 180° U-bend to the double offset U-bend, the effect of turbulence on the motion of sand particles maximizes in the downstream section and results in erosive wear in Zones A, B, and C. Moreover, the gravitation force direction is parallel to the flow, and the sand particles are more likely to alter the path followed and fall down. A typical sand trajectory is simulated, and no significant rebounding takes place. It can be observed from sand tracks that particles travel in a direction close to the outer wall in both elbow configuration and continuing downstream.

**Figure 15:**
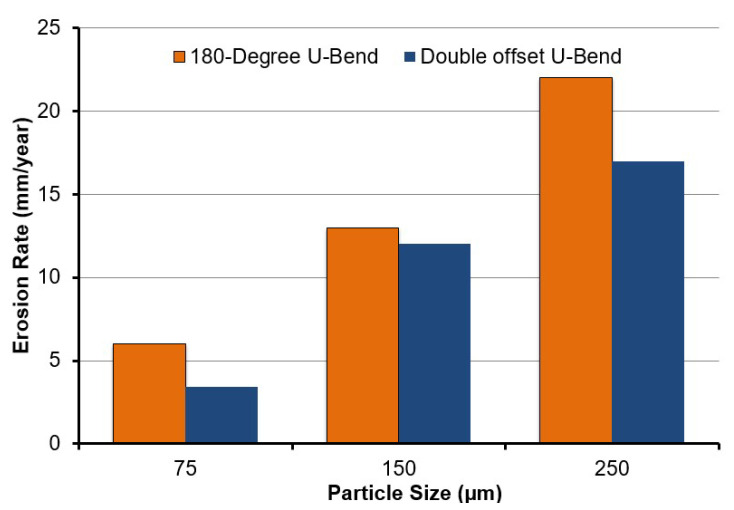
Maximum erosion rate in 180° U-bend and double offset U-bend for three different particle sizes.

The erosion rate is one of the most vital measures of flow-changing devices (i.e., elbow) lifetime in erosive flow conditions. Although the maximum erosion rate was seen to accrete with the increase in sand size for both the 180° U-bend and double offset U-bend for all evaluated cases, the double offset U-bend is less prone to erosive wear as shown in Figure 15. Notwithstanding this, the double offset U-bend proves its worth, as the highest rate of erosion is 1.23 times less than that of the 180° U-bend in the worst-case scenario.

## 4. Conclusions

A liquid-solid erosion simulation of the 180° U-bend and double offset U-bend has been employed to predict the erosive wear rate and distribution on both types of bend for sand-water flow conditions. Several simulations were performed to understand erosion patterns and trajectories of sand particles. Conforming to the numerical results, the following conclusions can be derived:

1. Erosive wear is the maximum at the outer curvature of the 180° U-bend and double offset U-bend for all evaluated cases. The maximum erosion area occurs between curvature angles of 150° and 180°. Additionally, the double offset U-bend proves its worth, as the highest wear rate is 1.23 times less than that of the 180° U-bend in the worst-case scenario.
2. The erosion rate of the double offset U-bend was nearly 8.58 times less than 180° U-bend for the fluid velocity of 2 m/s and 1.82 times less for 4 m/s fluid velocities. The maximum erosion rate of double offset U-bend was reduced by 120% compared to the 180° U-bend for 6 m/s in liquid-solid flow.
3. The 180° U-bend can be replaced with a double offset U-bend to slow down pipe erosion, especially for inverse orientation. Since many hydrocarbon and mineral processing plants require sand particle transportation, the double offset U-bend elbow appears to be a worthwhile alternative.
4. The formation of an erosive wear pattern at the double offset U-bend and 180° U-bend is explained through the sand particle tracking. With the change of configuration from the 180° U-bend to the double offset U-bend, the effect of turbulence on the motion of sand decreases as the flow approaches downstream and results in less erosive wear in the double offset U-bend.

## Figures and Tables

**Table 1:** Summary of simulation cases.

Case	Fluid	Orientation	Sand Size (μm)	Bend Type	Flow Velocity (m/s)	Erodent Flow Rate (kg/s)
1	A	H–V upward	300	180°	45.72	1
2	W	H–V upward	200	180°	10	0.2
3	W	V-V	450	180°	2	0.3
4	W	V-V	450	180°	4	0.3
5	W	V-V	450	180°	6	0.3
6	W	V-V	450	Double offset	2	0.3
7	W	V-V	450	Double offset	4	0.3
8	W	V-V	450	Double offset	6	0.3
9	W	V-V	75	180°	6	0.3
10	W	V-V	150	180°	6	0.3
11	W	V-V	250	180°	6	0.3
12	W	V-V	75	Double offset	6	0.3
13	W	V-V	150	Double offset	6	0.3
14	W	V-V	250	Double offset	6	0.3

## Data Availability

The data presented in this study are available upon request from the corresponding author.
